# How many specimens make a sufficient training set for automated three-dimensional feature extraction?

**DOI:** 10.1098/rsos.240113

**Published:** 2024-06-19

**Authors:** James M. Mulqueeney, Alex Searle-Barnes, Anieke Brombacher, Marisa Sweeney, Anjali Goswami, Thomas H. G. Ezard

**Affiliations:** ^1^ School of Ocean & Earth Science, National Oceanography Centre Southampton, University of Southampton Waterfront Campus, Southampton, UK; ^2^ Department of Life Sciences, Natural History Museum, London, UK

**Keywords:** deep learning, data augmentation, image segmentation, planktonic foraminifera, feature extraction

## Abstract

Deep learning has emerged as a robust tool for automating feature extraction from three-dimensional images, offering an efficient alternative to labour-intensive and potentially biased manual image segmentation methods. However, there has been limited exploration into the optimal training set sizes, including assessing whether artficial expansion by data augmentation can achieve consistent results in less time and how consistent these benefits are across different types of traits. In this study, we manually segmented 50 planktonic foraminifera specimens from the genus *Menardella* to determine the minimum number of training images required to produce accurate volumetric and shape data from internal and external structures. The results reveal unsurprisingly that deep learning models improve with a larger number of training images with eight specimens being required to achieve 95% accuracy. Furthermore, data augmentation can enhance network accuracy by up to 8.0%. Notably, predicting both volumetric and shape measurements for the internal structure poses a greater challenge compared with the external structure, owing to low contrast differences between different materials and increased geometric complexity. These results provide novel insight into optimal training set sizes for precise image segmentation of diverse traits and highlight the potential of data augmentation for enhancing multivariate feature extraction from three-dimensional images.

## 1. Introduction

Three-dimensional imaging techniques, such as X-ray micro-computed tomography (micro-CT), have revolutionized the characterization of both internal and external structures of diverse objects. With the ability to generate high-resolution images, researchers can visualize and quantify intricate three-dimensional features with wide-ranging applications [1,2]. However, objective extraction of these features remains a major challenge.

Currently, the prevailing approach to extracting three-dimensional features from image data involves manual segmentation, which is a labour-intensive [3] and subjective process that lacks reproducibility [4]. This manual approach limits the study of a large number of samples and the exploration of complex hypotheses. As the acquisition of high-resolution scans has increased steadily [5,6], there is a pressing need to enhance the efficiency of this important processing step.

Machine learning techniques, particularly deep learning, and convolutional neural networks (CNNs) offer a promising solution for automating image segmentation. These methods can potentially accelerate this processing step to deliver accurate and repeatable results, while being accessible to various research fields [7,8]. However, despite their merits, a fundamental trade-off exists between the quantity of samples necessary for generating accurate neural networks and the time-consuming, subjective nature of manual segmentation for evaluation. The specific number of required samples is likely to vary across datasets and depends on the traits being extracted. Hence, there is an imperative to establish the minimum number of manually segmented specimens needed to train these neural networks and to understand how they vary in different trait extraction scenarios.

Strategies to generate training sets that reduce manual processing while maintaining performance are required. Smart interpolation, whereby pre-segmented slices and the volumetric image data are used to predict segmentation across the entire specimen, is one suggested approach [9,10], but this remains time-consuming for creating large training sets. Data augmentation, which artificially expands the size of the training set without collecting new data, can mitigate overfitting and bolster the accuracy of CNNs during training [11,12]. Consequently, data augmentation techniques may allow smaller training sets to achieve equivalent accuracy levels of their larger counterparts.

**Figure 1.**
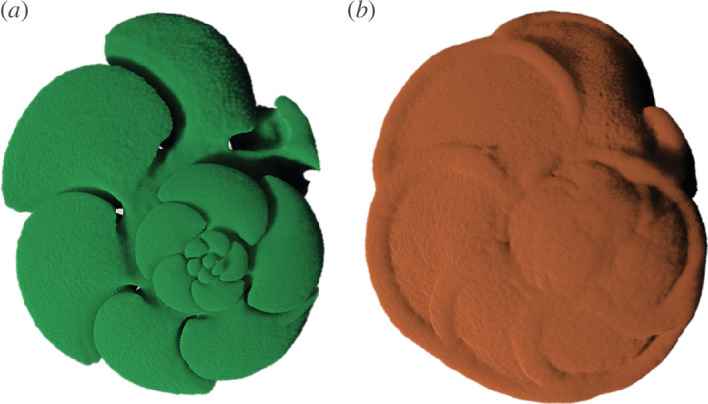
Three-dimensional models of the (*a*) internal and (*b*) external structure of planktonic foraminifera generated using manual segmentation in Dragonfly v. 2021.3 (Object Research Systems, Canada).

In this study, we aim to determine the minimum number of images needed to train a neural network to produce segmentation data that is statistically indistinguishable from manually generated data. To do so, we use a dataset of computed tomography (CT) scans of planktonic foraminifera from the genus *Menardella*. We assess the efficacy of each training set in extracting volumetric and shape data for the external calcite and internal chamber space of selected specimens (see figure 1).

Additionally, we introduce a novel three-dimensional data augmentation technique to bolster training sets by generating six different orientations of each specimen through rotation. This comparison serves to assess how data augmentation strategies can improve training sets to achieve accurate and efficient three-dimensional feature extraction.

## 2. Material and methods

### 2.1. Data collection

Fifty planktonic foraminifera, comprising 4 *Menardella menardii*, 17 *Menardella limbata*, 18 *Menardella exilis* and 11 *Menardella pertenuis* specimens, were used in our analyses (electronic supplementary material, figures S1 and S2). The taxonomic classification of these species was established based on the analysis of morphological characteristics observed in their shells. In this context, all species are characterized by lenticular, low trochospiral tests with a prominent keel [13]. Discrimination among these species is achievable, as *M. limbata* can be distinguished from its ancestor, *M. menardii*, by having a greater number of chambers and a smaller umbilicus. Moreover, *M. exilis* and *M. pertenuis* can be discerned from *M. limbata* by their thinner, more polished tests and reduced trochospirality. Furthermore, *M. pertenuis* is identifiable by a thin plate extending over the umbilicus and possessing a greater number of chambers in the final whorl compared with *M. exilis* [13].

The samples containing these individuals and species spanned 5.65 to 2.85 Ma [14] and were collected from the Ceara Rise in the Equatorial Atlantic region at Ocean Drilling Program (ODP) Site 925, which comprised Hole 925B (4°12.248' N, 43°29.349' W), Hole 925C (4°12.256' N, 43°29.349' W) and Hole 925D (4°12.260' N, 43°29.363' W). See Curry *et al*. [15] for more details. This group was chosen to provide inter- and intra-specific species variation, and to provide contemporary data to test how morphological distinctiveness maps to taxonomic hypotheses [16].

The non-destructive imaging of both internal and external structures of the foraminifera was conducted at the µ-VIS X-ray Imaging Centre, University of Southampton, UK, using a Zeiss Xradia 510 Versa X-ray tomography scanner. Employing a rotational target system, the scanner operated at a voltage of 110 kV and a power of 10 W. Projections were reconstructed using Zeiss Xradia software, resulting in 16-bit greyscale .tiff stacks characterized by a voxel size of 1.75 μm and an average dimension of 992 × 1015 pixels for each two-dimensional slice.

### 2.2. Generation of training sets

**Figure 2.**
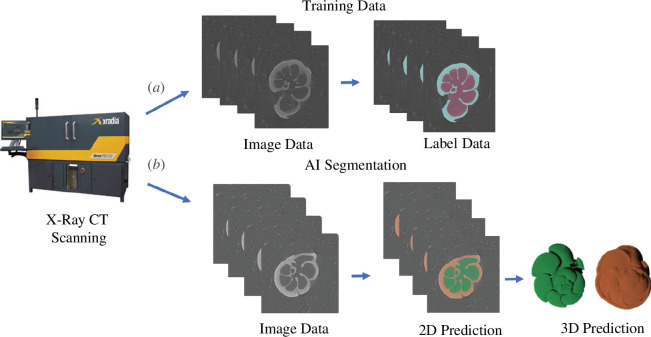
Workflow for producing training data and applying a deep CNN to perform automated image segmentation. The workflow includes (*a*) the creation of training data for the input into Biomedisa and (*b*) an example application of the trained CNN to automate the process of generating segmentation (label) data.

We extracted the external calcite and internal cavity spaces from the micro-CT scans of the 50 individuals using manual segmentation within Dragonfly v. 2021.3 (Object Research Systems, Canada). This step took approximately 480 min per specimen (24 000 min total) and involved the manual labelling of 11 947 two-dimensional images. Segmentation data for each specimen were exported as multi-label (three labels: external, internal and background) 8-bit multipage .tiff stacks and paired with the original CT image data to allow for training (see figure 2).

The 50 specimens were categorized into three distinct groups (electronic supplementary material, table S1): 20 training image stacks, 10 validation image stacks and 20 test image stacks. From the training image category, we generated six distinct training sets, varying in size from 1 to 20 specimens (see table 1). These were used to assess the impact of training set size on segmentation accuracy, as determined through a comparative analysis against the validation set (see §2.3).

**Table 1.** Design of the training sets detailing the name of the training set, the data type (original or augmentation), the number of specimens in each training set, the number of two-dimensional images available for training and the approximate time to perform the manual segmentation to create the training set.

name of training set	data type	number of specimens	no. of foreground images (XY)	approx. duration of segmentation (mins)
T0_AI_1_Images	original	1	241	480
T1_AI_2_Images	original	2	390	960
T2_AI_4_Images	original	4	1028	1920
T3_AI_8_Images	original	8	2187	3840
T4_AI_16_Images	original	16	3571	7680
T5_AI_20_Images	original	20	4251	9600
Aug_T0_AI_1_Images	augmentation	1	4480	480
Aug_T1_AI_2_Images	augmentation	2	8812	960
Aug_T2_AI_4_Images	augmentation	4	18 120	1920
Aug_T3_AI_8_Images	augmentation	8	36 398	3840
Aug_T4_AI_16_Images	augmentation	16	71 100	7680
Aug_T5_AI_20_Images	augmentation	20	88 432	9600

**Figure 3.**
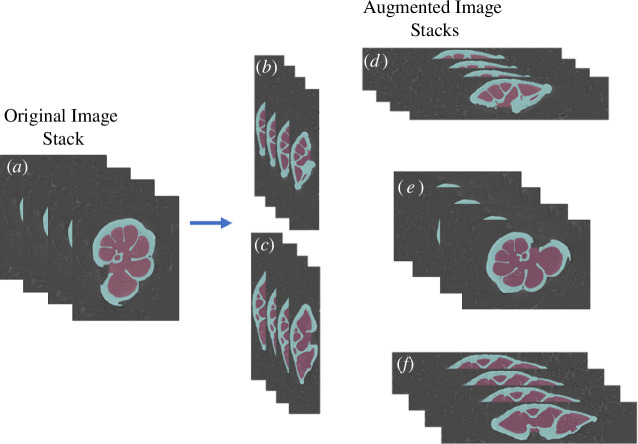
Rotation of the original image to form the new augmentation training data. The original image in an (*a*) *xyz* orientation is rotated into five other three-dimensional planes: (*b*) *yzx*, (*c*) *zyx*, (*d*) *xzy*, (*e*) *yxz* and (*f*) *zxy* orientations. These are then all paired together and used in training.

From the initial six training sets, we created six additional training sets through data augmentation using the NumPy library [17] in Python. This augmentation method was chosen for its simplicity and accessibility to researchers with limited computational expertise, as it can be easily implemented using a straightforward batch code. This augmentation process entailed rotating the original images five times (the maximum amount permitted using this method), effectively producing six distinct three-dimensional orientations per specimen for each of the original training sets (see figure 3). The augmented training sets comprised between 6 and 120 .tiff stacks (see table 1).

### 2.3. Training the neural networks

CNNs were trained using the offline version of Biomedisa [10], which utilizes a three-dimensional U-Net architecture [18]—the primary model employed for image segmentation [19]—and is optimized using Keras with a TensorFlow back end. We used patches of size 64 × 64 × 64 voxels, which were then scaled to a size of 256 × 256 × 256 voxels. This scaling was performed to improve the network’s ability to capture spatial features and mitigate potential information loss during training. We trained three networks for each of the training sets to check the extent of stochastic variation in the results [20].

To train our models in Biomedisa, we used a stochastic gradient descent with a learning rate of 0.01, a decay of 1 × 10^−6^, momentum of 0.9 and Nesterov momentum enabled. A stride size of 32 pixels and a batch size of 24 samples per epoch were used alongside an automated cropping feature, which has been demonstrated to enhance accuracy [21]. The training of each network was performed on a Tesla V100S-PCIE-32GB graphics card with 30 989 MB of available memory. All the analyses and training procedures were conducted on the high-performance computing system at the Natural History Museum, London.

To measure network accuracy, we used the Dice similarity coefficient (Dice score), a metric commonly used in biomedical image segmentation studies [22,23]. The Dice score quantifies the level of overlap between two segmentations, providing a value between 0 (no overlap) and 1 (perfect match). For two segmentations, X and $X^{\prime }$ consisting of *n* labels, the Dice score is defined as


$$Dice=\frac{2\sum_{i=1}^{n}\left(X_{i}\cap X_{i}^{\prime }\right)}{\left|X\right|+\left|X^{\prime }\right|},$$

where $\left|X\right|$ and $\left|X^{\prime }\right|$ are the total number of voxels of each segmentation, respectively, and $X_{i}$ is the subset of voxels of X with label *i*.

We conducted experiments to evaluate the potential efficiency gains of using an early stopping mechanism within Biomedisa. After testing a variety of epoch limits, we opted for an early stopping criterion set at 25 epochs, which was found to be the lowest value as to which all models trained correctly for every training set. By ‘trained correctly’ we mean if there is no increase in Dice score within a 25-epoch window, the optimal network is selected, and training is terminated. To gauge its impact of early stopping on network accuracy, we compared the results obtained from the original six training sets under early stopping with those obtained on a full run of 200 epochs.

### 2.4. Evaluation of feature extraction

We used the median accuracy network from each of the 12 training sets to produce segmentation data for the external and internal structures of the 20 test specimens. The median accuracy was selected as it provides a more robust estimate of performance by ensuring that outliers had less impact on the overall result. We then compared the volumetric and shape measurements from the manual data with those from each training set. The volumetric measurements were total volume (comprising both external and internal volumes) and percentage calcite (calculated as the ratio of external volume to internal volume, multiplied by 100).

To compare the shape, mesh data for the external and internal structures was generated from the segmentation data of the 12 training sets and the manual data. Meshes were decimated to 50 000 faces and smoothed before being scaled and aligned using Python and generalized Procrustes surface analysis [24], respectively. Shape was then analysed using the landmark-free morphometry pipeline, as outlined by Toussaint *et al*. [25]. We used a kernel width of 0.1 mm and noise parameter of 1.0 for both the analysis of shape for both the external and internal data, using a Keops kernel (PyKeops; https://pypi.org/project/pykeops/) as it performs better with large data [25]. The analyses were run for 150 iterations, using an initial step size of 0.01. The manually generated mesh for the individual st049_bl1_fo2 was used as the atlas for both the external and internal shape comparisons.

## 3. Results

### 3.1. Early stopping versus 200 epochs

**Figure 4.**
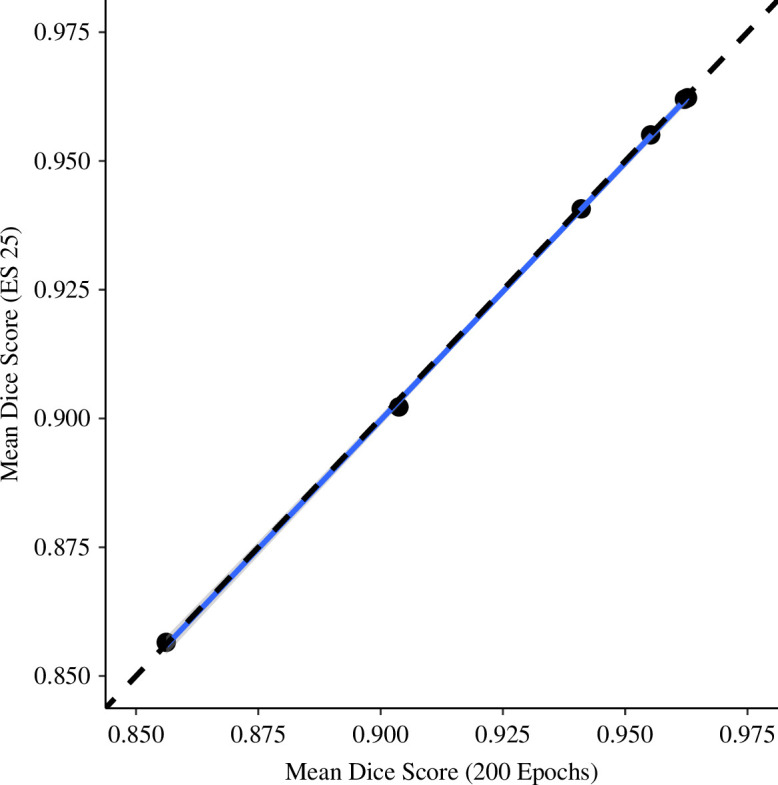
Comparison of Dice scores obtained from early stopping within 25 epochs and a full run of 200 epochs for each of the six original image training sets shows no statistical difference. The correlation coefficient (*r^2^*) between the two sets of scores displayed is 0.9997.

A likelihood ratio test found no detectable difference between the observed correlation of the six original training sets under early stopping within 25 epochs and the full run of 200 epochs (analysis of variance, *F*_4, 5_ = 0.0424, *p* > 0.05), with a strong correlation between the early stopping and full run values (see figure 4; *R* ^2^ = 0.9997, *p* < 0.001). Consequently, we report results using the early stopping criterion for all subsequent tests.

### 3.2. Network accuracy

**Figure 5.**
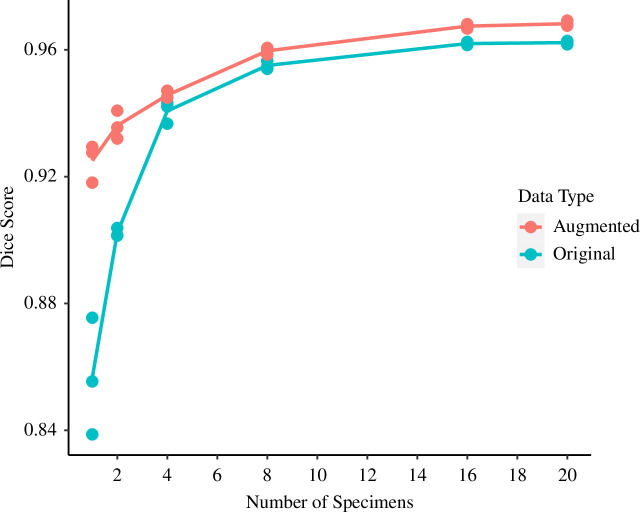
Comparison of segmentation accuracy for Biomedisa automated segmentation using average Dice scores calculated for validation data generated from 10 specimens. The plot shows increasing the training set size and implementing data augmentation improves network accuracy. All models were trained using early stopping within 25 epochs.

The Dice scores of both the original and augmented datasets increased with the number of training images (figure 5; electronic supplementary material, table S2). The improvement in accuracy from 1 specimen to 20 specimens was 12.34% and 4.67% for the original and augmented datasets, respectively. Most of this improvement occurred between training sets of one to eight specimens. Dice scores varied across all training sets (analysis of variance, *F*_11, 24_ = 92.84, *p* < 0.001). The augmented training data resulted in higher mean Dice scores compared with their original counterparts, especially at lower specimen numbers (figure 5). Increasing specimen numbers increased the Dice score for the augmented data (*β* = 0.314 on the scale of the logit link function from a generalized linear model with a quasibinomial error structure, s.e. = 0.027, *p* < 0.001), but did so faster for the original data (*β* = 0.211 on the scale of the logit link function, s.e. = 0.031, *p* < 0.001) albeit from a much lower initial baseline (*β* = −0.633 on the scale of the logit link function, s.e. = 0.0059, *p* < 0.001). The model explains 96% of the variation in the data.

### 3.3. Volumetric comparison

**Figure 6.**
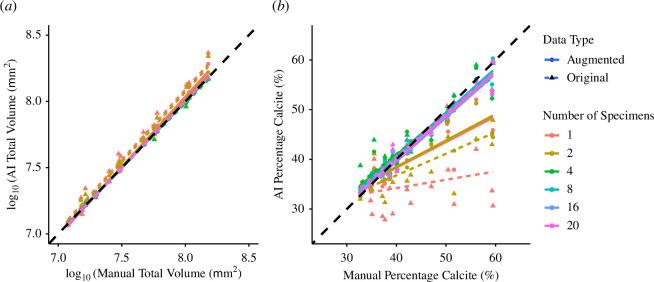
Comparison of (*a*) total volumes (internal and external combined) and (*b*) percentage calcite obtained from manual segmentation compared with those obtained using AI segmentation using the different training sets consisting of different numbers of training images. The predictions of total volume are generally more accurate than percentage calcite.

We identified a significant overall correlation between the manually generated and network-generated total volumes for the 20 test images (*F*_1, 216_ = 51 223, *p* < 0.001; figure 6; electronic supplementary material, table S3), but uncovered significant differences in the degree of these correlations across training sets (*F*_11, 216_ = 19.660, *p* < 0.001). Here, the *R* ^2^ values, serving as indicators of goodness of fit, spanned from 0.9505 for the training set with 1 specimen to 0.9998 for that containing 20 specimens with data augmentation—a difference of 0.0493.

For percentage calcite, the correlation between the manually generated and network-generated values was also significant (*F*_1, 216_ = 1093.2, *p* < 0.001), and again significant differences were noted in the degree of correlation across training sets (*F*_11, 216_ = 21.367, *p* < 0.001). Notably, the deviation in the gradients from a perfect fit was more pronounced for the lower training sets for percentage calcite than for the total volumes. This difference was reflected in the *R* ^2^ values, which ranged from 0.0608 for the training set with 1 specimen to 0.9607 for the 20-specimen set with image augmentation—a much greater difference of 0.8999.

### 3.4. Shape comparison

We expanded the analysis to compare shape estimates between the manually derived data and the network data for both external and internal structures. In total, 16 530 control points were generated for the external structure, while 17 325 control points were generated for the internal structure. These data points were subsequently reduced to five principal axes which display 100% of the total variation through non-linear kernel principal component analysis (kPCA) [26], using 1000 iterations. This dimensionality reduction technique works by allowing the separation of nonlinear data by making use of kernels and projecting data into higher dimensional space where it becomes linearly separable.

**Figure 7.**
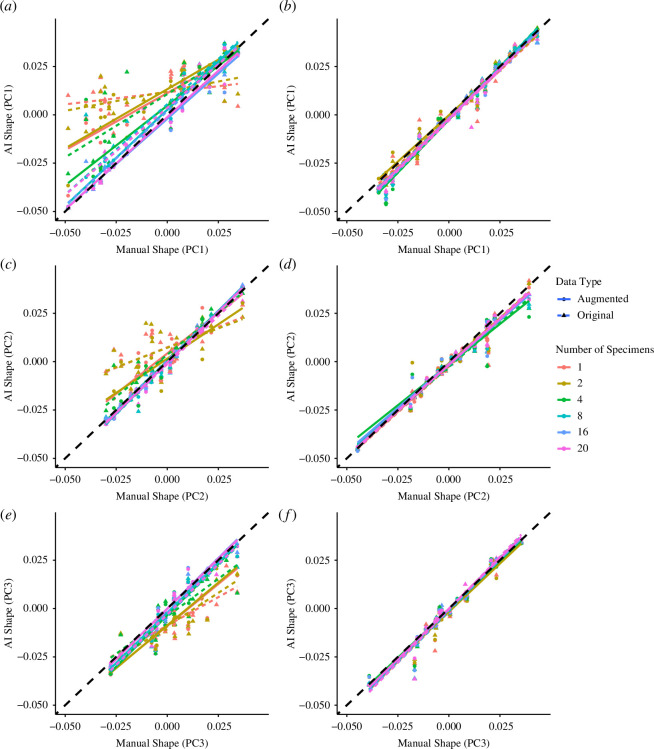
Comparison of shape for the internal (*a, c* and *e*) and external (*b, d* and *f*) structures across PC axes 1 (*a, b*), 2 (*c, d*) and 3 (*e, f*) obtained from manual segmentation compared with those obtained using AI segmentation using the different training sets consisting of different numbers of training images. The figure demonstrates that external calcite is easier to predict than the internal chamber space.

Both the internal and external shape data exhibited strong and statistically significant correlations between the manually generated and network-generated shape values across all three axes, indicating consistency in their shape estimates (figure 7; table 2; electronic supplementary material, tables S4 and S5). In the analysis of the external structure, the high correlation between the manually and network-generated data across all training sets was maintained (table 2). When performing kPCA on the external shape results, we found that PC1 controlled 36.4% of the total variation, followed by PC2 with 19.2%, and PC3 with 17.9%, resulting in the first three axes controlling 73.5% of the total variation (electronic supplementary material, table S6). By contrast, the internal structures displayed substantial variations across the training sets as the number of images used increased from 1 to 20 across all three axes (table 2), indicating differences among the accuracy of different training sets. In this analysis of internal structure, PC1 accounted for 37.6% of the total variation, followed by PC2 with 19.7% and PC3 with 18.1%, resulting in the first three axes controlling 75.4% of the total variation (electronic supplementary material, table S7). Owing to the high proportion of variance controlled by these PC axes, we use these for our primary comparisons.

**Table 2.** There was no quantitative difference between manually generated and network-generated shape estimates for internal and external structures (left as *F* statistics on 1 and 216 degrees of freedom), but clear differences in predictive ability between internal and external shape estimates as the numbers of training images increase (right as *F* statistics on 11 and 216 degrees of freedom). *F* statistics are presented to our significant figures; values in bold indicate *p *< 0.05 whereas non-bold is *p *> 0.05.

	*F*-statistic between manual and network predictions		*F*-statistic among different numbers of training images	
axis	internal	external	internal	external
PC1	**548.4**	**7918**	**5.442**	0.5695
PC2	**1002**	**3474**	**3.893**	0.2491
PC3	**1208**	**3886**	**6.997**	0.3501

## 4. Discussion

In this study, we observed a positive correlation between network accuracy and the quantity of training images, with data augmentation proving an effective tool for enhancing performance, especially for smaller training sets. External structures were extracted relatively accurately in the smaller training sets, unlike the internal structures, and size was more straightforward to extract than shape. Noting that some traits will always be more difficult to extract accurately than others, we discuss key concepts for the ongoing implementation of automated feature extraction in the biological sciences.

### 4.1. Impact of early stopping

The early stopping feature substantially reduced training time while effectively preserving network accuracy (electronic supplementary material, table S2). These qualities made the early stopping feature particularly valuable for larger training sets, and when applying data augmentation techniques.

Beyond improving time efficiency, the integration of early stopping features also serves to mitigate generalization errors and overfitting [27,28]. As a result, the application of early stopping features is widely applied when training deep learning models [29,30]. Our findings strongly endorse the use of these early stopping features in future research using three-dimensional image segmentation.

### 4.2. Impacts of training set size and data augmentation

Our findings reaffirm the principle that expanding the training set leads to the production of better deep learning models [31,32], albeit with diminishing returns as accuracy approaches 100% [33]. The expansion of available training data plays a crucial role in reducing generalization error and thus, in facilitating the achievement of optimal accuracy levels [34]. This is reflected in the stability of accuracy observed across epochs in larger training datasets, which are less affected by noise (electronic supplementary material, figures S3–S14).

Importantly, the minimum number of training images to achieve accurate results and the degree to which increasing the training set size enhances network accuracy remains task-specific and context-dependent [35]. The choice of the network architecture may vary in its suitability for segmenting specific material or structures [36], meaning training sets must carefully be selected. Notably, in this study, only a small number of individuals were required to achieve high accuracy scores, with eight specimens being required to achieve the goal of 95% accuracy (with and without augmentation) when comparing the manually and network-generated data. This achievement can be attributed to the wealth of two-dimensional slices per specimen and the ready availability of high-quality segmentation data. Without these attributes, a larger number of individuals for training would probably be necessary, resulting in delayed increases in accuracy. Consequently, the generation of high-quality training data emerges as a factor more crucial than its sheer quantity [37], with any errors or inconsistencies within the training set likely to manifest in the deep learning models.

Data augmentation is a valuable tool for enhancing network accuracy, addressing limitations stemming from insufficient training data availability. Our results strongly support that data augmentation can effectively boost model accuracy [38,39]. Although variable by feature, we have demonstrated that it is possible to train a network with as few as one or two specimens to extract segmentation data almost as accurately as eight specimens using this augmentation approach. As a result, fewer images are needed for training and thus we can significantly reduce the time spent manually segmenting. Moreover, its application in this context is straightforward and universally applicable to three-dimensional image datasets.

### 4.3. Extracting phenotypic traits

The methodology presented in this study represents a significant advancement in the extraction of phenotypic traits from organisms such as planktonic foraminifera, offering notable improvements in processing times and repeatability compared with other established methods [40,41]. Our approach exhibits remarkable efficiency, culminating in the generation of results for a single specimen in approximately a minute, thus facilitating the execution of large-scale studies with unprecedented speed. Moreover, our methodology excels in repeatability, as it mitigates the impact of fluctuations in thresholding or greyscale values, ensuring more consistent and reliable data extraction. Crucially, our approach extends the scope of trait extraction beyond percentage calcite, encompassing additional parameters such as overall size (internal + external volumes) and shape, which remain elusive with alternative methodologies. This multi-faceted advancement underscores the versatility and efficacy of our approach in unravelling the intricacies of foraminiferal morphology.

Our results suggest extracting the internal structure of planktonic foraminifera poses a greater challenge compared with extraction of the external structure, evidenced in the measures of percentage calcite and the shape of the internal structure (see figures 6*b* and 7). This discrepancy in difficulty can probably be attributed to material homogeneity, which is reflected in low contrast differences within CT scans [42,43]. This challenge is particularly prominent in the case of planktonic foraminifera, where the internal structure contains sedimentary infill and nannofossil ooze with densities similar to external calcite [44,45]. The quantity of this infill is likely to vary among specimens, ranging from abundant to absent, while the presence of external calcite remains consistently detectable. Consequently, to alleviate the impact of irrelevant features, such as sediment infill, on trait extraction, we need training sets that encompass a broader array of images, representing a fuller sample of the entire image population compared with datasets without these elements. This can be achieved by either increasing the size of the training set or employing data augmentation techniques, thereby facilitating more accurate predictions.

Further enhancements in the accuracy of selected networks and mitigations of irrelevant features can be achieved through the application of post-processing tools. These tools facilitate the removal of connected components and the application of smoothing methods to eliminate noise from deep learning outputs [46,47], both of which hold the potential to yield more precise segmentation data.

### 4.4. Implications for future studies

There is a growing demand in the field of phenomics for the integration of machine learning techniques to perform image segmentation, driven by the increasing abundance of available data [48]. Unlike manual segmentation, which is inherently prone to observer bias [4], deep learning methods offer a robust means to ensure greater consistency across measurements [49], facilitating efficient and scalable comparative studies. The complexity and size of phylogenies [50] and birth–death models [51] are only getting larger, with the use of continuous traits [52] and geometric morphometric approaches [53] now commonplace. Thus, there is a need to develop morphometric approaches that can keep pace with these advances.

The application of automated segmentation and the data augmentation techniques applied here can be used universally to improve data acquisition. They integrate seamlessly with widely used segmentation software like Dragonfly, Avizo/Amira, ImageJ and MITK, so can be incorporated into existing workflows. They also accommodate various input sources, encompassing images obtained through X-ray synchrotron microscopy [54], soft microscopy [55] and magnetic resonance imaging [56]. This versatility empowers the workflow to conduct large-scale analyses efficiently and with a high level of accuracy within reasonable timeframes. With the provision of suitable input data [57], these trained networks become accessible to anyone for segmentation, regardless of their expertise in the morphology of the selected specimen.

The ability to extract different three-dimensional features using deep learning remains likely to be context specific, with a variety of features such as the presence of artefacts and irregularities [58], broken or incomplete specimens [59] and low-contrast images [47] each requiring individual solutions to overcome. Improvement in the quality of image data acquisition and selection of specimens can aid in mitigating some of these factors [6]; however, these must coincide with high-quality training sets.

The degree of tolerable error is also influenced by the quantification required. Here, our results demonstrate that extracting the total volume was easier than percentage calcite and shape. The variation in total volume is much greater and is less complicated to measure, and thus is less confounded by inaccurate measurements than more plastic traits. The landmark-free method applied here, for instance, requires the generation of meshes, and scaling and alignment of the data, which further produces error. Researchers should thus consider their intended measurements when building training data and then during the actual training of their neural networks.

## 5. Conclusion

We provide compelling evidence that leveraging deep learning for automated segmentation yields equivalent results to manual segmentation when extracting three-dimensional features from images and also drastically reduces the time required. Data augmentation emerged as a powerful tool, elevating network accuracy and enhancing the efficacy of smaller training sets. However, it is imperative to acknowledge that the accuracy of feature extraction is contingent on the specific traits targeted, with eight specimens being required here to extract all of the desired traits within levels of 95% accuracy in this case study. The observed differences for internal and external traits extracted underscore the crucial need for thoughtful consideration when training deep learning networks for specific applications.

## Data Availability

All computer code and final raw data used in the paper is available at [60]. All other data is available in the Dryad Repository [61]. Supplementary material is available online [62].
